# Supplementary material for: HTO/Cellulose Aerogel for Rapid and Highly Selective Li+ Recovery from Seawater
Source: Molecules. 2021 Jul 2;26(13):4054. doi: 10.3390/molecules26134054 (PMC8272140; doi:10.3390/molecules26134054)
Supplement: Supplementary file 1 [file molecules-26-04054-s001.zip › molecules-1259641-supplementary.pdf]

Supplementary Information

HTO/Cellulose Aerogel for Rapidly and Highly Selective Li^+ Recovery from Seawater

Hongbo Qian [1], Shaodong Huang [1], Zhichen Ba [1], Wenxuan Wang [1], Feihan Yu [1], Daxin Liang [1,*], Yanjun Xie [1], Yonggui Wang [1] and Yan Wang [2,*]

[1] Key Laboratory of Bio-based Material Science and Technology, Ministry of Education, Northeast Forestry University, Harbin 150040, P. R. China; daxin.liang@nefu.edu.cn

[2] Harbin Center for Disease Control and Prevention, Harbin 150056, P. R. China;

[*] Correspondence: daxin.liang@nefu.edu.cn (D. Liang); 18745015921@126.com (Y. Wang)

Experimental

To explore the Li^+ adsorption behavior in HTO powder and HTO in CA, in accordance with the following equations, using Eqs. (1) and (2), respectively.

$$\ln(q_e - q_t) = \ln q_e - k_1 t \quad (1)$$

$$\frac{t}{q_t} = \frac{1}{k_2 q_e^2} + \frac{t}{q_e} \quad (2)$$

The results of adsorption were applied to the Langmuir and Freundlich models to investigate the Li^+ adsorption isotherm, using Eqs. (3) and (4), respectively.

$$\frac{C_e}{q_e} = \frac{C_e}{q_m} + \frac{1}{q_m \times K_L} \quad (3)$$

$$\ln q_e = \ln K_F + \frac{1}{n}\ln C_e \quad (4)$$

Table S1. Pseudo-first-order and Pseudo-second-order

Material	Pseudo-first-order			Pseudo-second-order		
	q_e ($mg\cdot g^{-1}$)	$k_1\times10^{-3}$ (min^{-1})	r^2	Qe ($mg\cdot g^{-1}$)	$k_1\times10^{-3}$ (min^{-1})	r^2
HTO powder	13.52	1.15	0.938	3.52	28.76	0.995
HTO/CA	3.77	1.40	0.796	17.38	28.35	0.999

Table S2. Langmuir model and Freundlich model

Temperature	Langmuir isotherm			Freundlich isotherm		
	q_m ($mg \cdot g^{-1}$)	k_1	r^2	k_2	n	r^2
25℃	26.75	3.82	0.999	18.29	18.29	0.765
35℃	28.61	2.63	0.999	17.85	7.40	0.732
45℃	28.53	3.41	0.999	18.96	8.29	0.753
